# Explosive cervical lymph node recurrence following two-field lymphadenectomy in pT1b (SM2) middle esophageal squamous cell carcinoma with INFb pattern and absent lymphovascular invasion: a case report

**DOI:** 10.3389/fonc.2026.1976165

**Published:** 2026-10-16

**Authors:** Jianpeng Che, Yao Wei, Wei Li, Linlin Ji

**Affiliations:** 1 Department of Thoracic Surgery, Weifang People’s Hospital, Weifang, China; 2 Department of Radiation Oncology, Weifang Traditional Chinese Hospital, Weifang, China

**Keywords:** case report, cervical lymph node recurrence, endoscopic submucosal dissection, esophageal squamous cell carcinoma, infiltrative growth pattern

## Abstract

**Background:**

In superficial esophageal squamous cell carcinoma (ESCC), clinical decisions are guided by submucosal invasion depth (200-μm cutoff) and lymphovascular invasion (LVI) status. Although the tumor infiltration (INF) pattern is an independent risk factor, it is often overlooked when planning surgical strategies and surveillance. We report a case highlighting the need to integrate INF classification into practice, with emphasis on cervical lymphatic drainage.

**Case Presentation:**

A 51-year-old man underwent endoscopic submucosal dissection (ESD) for middle thoracic ESCC. Histopathology revealed moderately differentiated SCC with submucosal invasion to 370 μm, staged as pT1b (SM2) (JES classification), with an INFb pattern, negative LVI, and negative margins. Because invasion depth exceeded 200 μm, radical esophagectomy was performed 14 days after ESD. Preoperative CT identified a tiny left 101L (cervical paraesophageal) lymph node, but this was clinically dismissed given the perceived low-risk features. The patient underwent standard two-field lymphadenectomy without adjuvant therapy. CT at 5 months was unremarkable; at 9 months, a 60×39 mm mass appeared in the left 101L region with tumor thrombus in the left internal jugular vein. Biopsy confirmed metastatic SCC. After cervical recurrence, the patient received concurrent chemoradiotherapy; the metastatic node showed partial response after 4 cycles, and further chemotherapy plus sequential immunotherapy is planned.

**Conclusion:**

Middle thoracic ESCC with an INFb pattern may carry a substantial risk of cervical lymph node metastasis, even in pT1b (SM2), LVI-negative tumors. When preoperative imaging reveals cervical nodes of uncertain significance, standard two-field dissection leaves a critical “blind spot.” 18F-FDG PET-CT should be performed in accordance with current staging guidelines, and the extent of lymphadenectomy should be individualized based on preoperative imaging findings. Intensified surveillance using cervical and thoracic thin-slice CT should be considered.

## 1 Introduction

Lymph node metastasis remains one of the most critical determinants of prognosis in esophageal cancer. For superficial esophageal squamous cell carcinoma (ESCC), treatment strategies have traditionally been shaped by two primary pathological parameters: the depth of submucosal invasion (with the 200-μm threshold serving as the key cutoff for endoscopic resection eligibility) and the presence or absence of lymphovascular invasion (LVI). Current guidelines generally recommend esophagectomy for tumors that invade beyond 200 μm into the submucosa, while lesions without LVI are often categorized as relatively low-risk for systemic dissemination. Nevertheless, even when these evidence-based protocols are strictly followed, a subset of patients experience unexpected regional recurrence, suggesting that additional risk factors are at play.

The tumor infiltration (INF) pattern—particularly the intermediate (INFb) and infiltrative (INFc) types—has emerged as a factor of growing interest in this context. INFc has been established as an independent risk factor for lymph node metastasis, distinct from both invasion depth and LVI status (1, 2), and some studies have also suggested a potential association between INFb and elevated metastatic risk (3). Despite accumulating evidence, the incorporation of INF classification into routine clinical decision-making remains limited. Moreover, the middle thoracic esophagus, by virtue of its unique embryologic origin and complex bidirectional lymphatic drainage network that includes ascending channels to the cervical region, carries a non-negligible risk of cervical lymph node metastasis—a risk that may be exacerbated when an infiltrative tumor front is present. In this report, we describe the complete clinical course of a patient with INFb-type, pT1b (SM2), LVI-negative ESCC of the middle thoracic esophagus who developed a massive ipsilateral cervical lymph node recurrence shortly after undergoing standard two-field lymphadenectomy. Through this case, we seek to demonstrate that the convergence of an infiltrative tumor phenotype and a mid-esophageal location can override the seemingly reassuring implications of negative LVI, even when invasion depth meets conventional surgical criteria.

## 2 Case description

### 2.1 Patient information and initial treatment

A 51-year-old male was admitted following the detection of a middle esophageal mucosal lesion on screening esophagogastroduodenoscopy. He reported no dysphagia, acid reflux, or other gastrointestinal symptoms. His medical history included coronary artery stenting, with long-term clopidogrel use (discontinued 3 weeks preoperatively), and a history of heavy smoking (30 pack-years). Preoperative endoscopy and endoscopic ultrasonography (EUS) revealed a rough, depressed mucosal lesion at 25–26 cm from the incisors, with an IPCL type B1 pattern (**Figures 1A, B**). EUS suggested the lesion was confined to the mucosal layer (T1a). A diagnostic ESD was performed (**Figures 1C, D**), achieving complete macroscopic resection.

**Figure 1:**
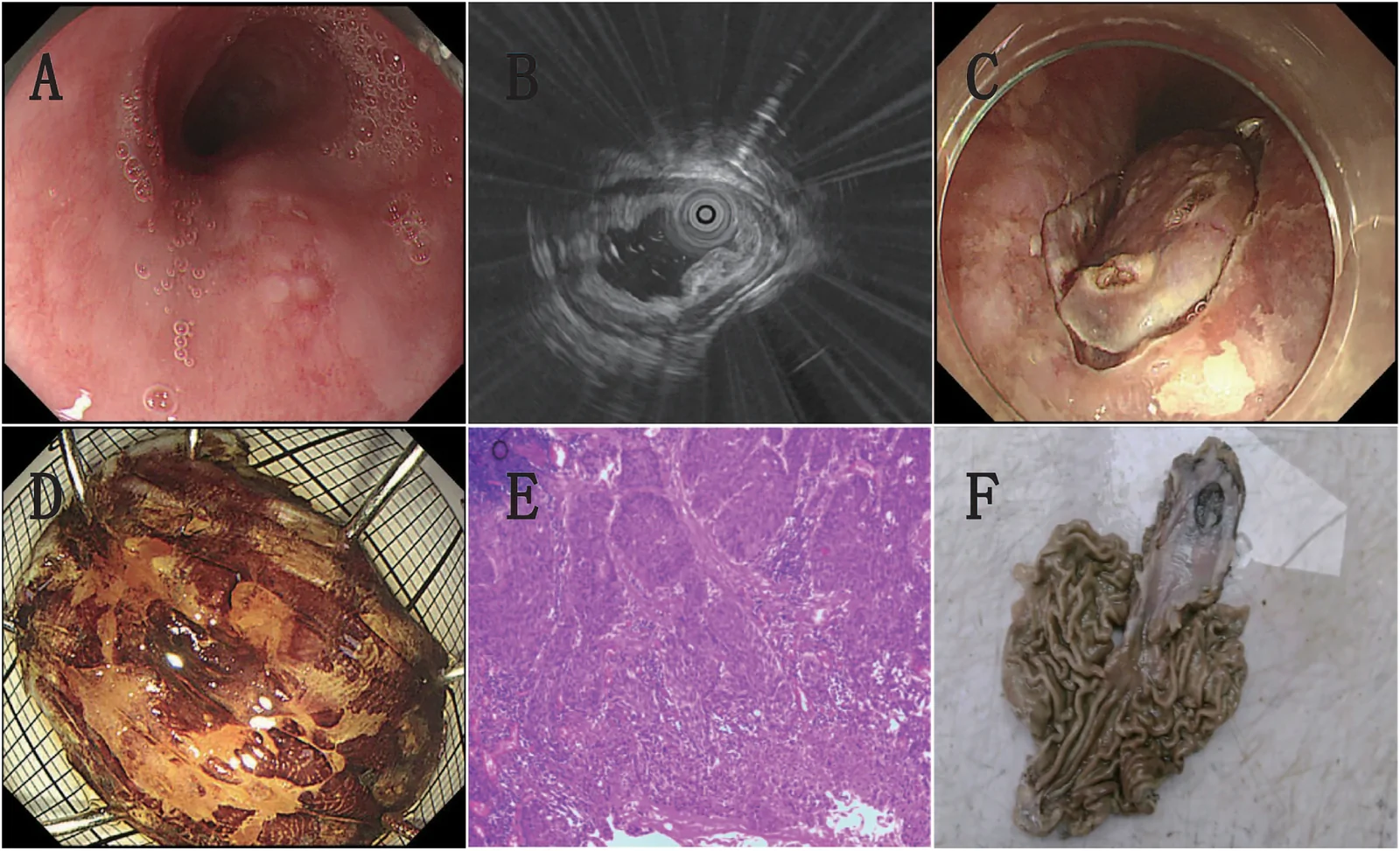
Endoscopic, macroscopic, and histopathological findings. **(A)** Preoperative esophagogastroduodenoscopy showing the primary lesion in the middle thoracic esophagus. **(B)** Endoscopic ultrasonography confirming the lesion confined to the mucosal layer (T1a). **(C)** Intraoperative view during endoscopic submucosal dissection (ESD). **(D)** Gross appearance of the ESD-resected specimen. **(E)** Histopathological examination of the ESD specimen (hematoxylin and eosin staining, low magnification) revealing moderately differentiated squamous cell carcinoma. **(F)** Gross view of the surgically resected esophagus following radical esophagectomy.

### 2.2 Post-ESD pathology and additional surgery

Histopathological examination of the resected specimen revealed moderately differentiated squamous cell carcinoma (**Figure 1E**), with a maximum invasion depth of 370 μm into the submucosa. According to the Japanese Esophageal Society (JES) classification, while the absolute depth exceeds the 200-μm threshold (classifying it as T1b), the relative one-third stratification corresponds to SM2 (middle third) given the typical esophageal submucosal thickness. The infiltration pattern was classified as INFb (mixed expansive and infiltrative growth). Immunohistochemical staining (D2-40, CD31) confirmed the absence of lymphatic and vascular invasion (Ly0, V0), and both horizontal and vertical resection margins were negative.

In accordance with guideline recommendations (invasion depth > 200 μm), the patient underwent thoracoscopic and laparoscopic radical esophagectomy 14 days after ESD to mitigate the risk of regional lymph node metastasis (**Figure 1F**). Crucially, preoperative contrast-enhanced CT of the neck and chest identified a tiny but suspicious lymph node in the left supraclavicular region (101L area) (**Figure 2A**). At that time, preoperative tissue diagnosis of this node was not considered. The tumor was judged to be superficial pT1b (SM2) with negative LVI, and the likelihood of cervical lymph node metastasis was therefore considered low. Functional imaging with ¹^8^F-FDG PET-CT was not performed, and no preoperative biopsy or fine-needle aspiration of the 101L node was attempted. Consequently, this imaging finding did not alter the surgical plan, and a standard thoracic and abdominal two-field lymphadenectomy was performed, with gastric conduit reconstruction and cervical esophagogastrostomy. Postoperative pathological examination of all resected lymph nodes (thoracic and abdominal) yield-ed negative results. The patient recovered uneventfully and received no adjuvant chemoradiotherapy after discharge.

**Figure 2:**
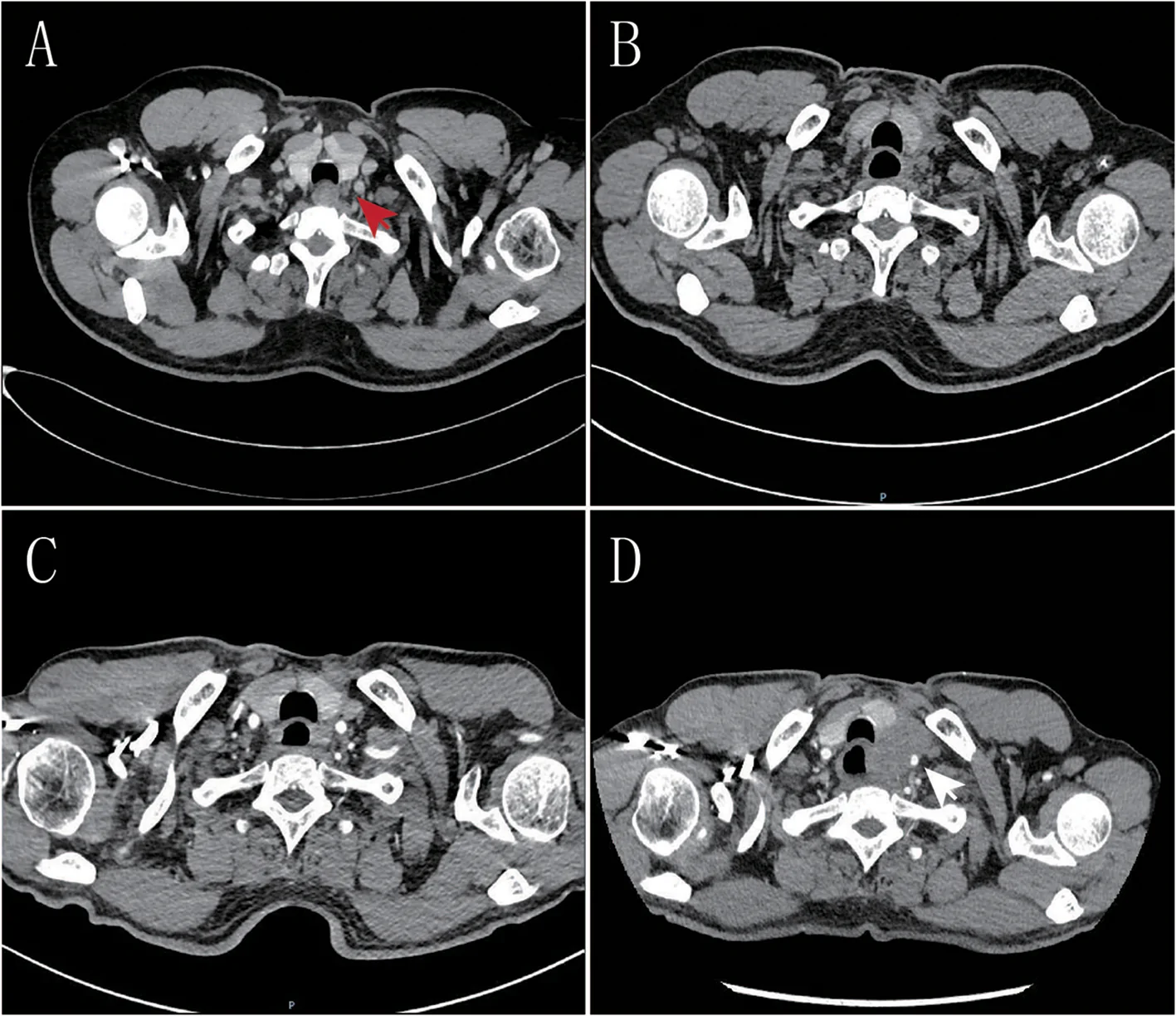
Dynamic CT evolution of cervical lymph node status. **(A)** Preoperative CT showing a tiny lymph node in the left 101L region (red arrow). **(B)** CT at 1 month post-surgery showing no significant change in this node. **(C)** CT at 5 months post-surgery revealing no definitive abnormality in the 101L area. **(D)** CT at 9 months post-surgery revealing a 60×39 mm irregular soft tissue mass in the left 101L region (white arrows), encasing the common carotid artery.

### 2.3 Follow-up, recurrence, and pathological confirmation

Postoperative surveillance consisted of combined cervical and thoracic thin-slice CT. At 1 month post-surgery (October 2025) (**Figure 2B**) and at 5 months post-surgery (February 2026) (**Figure 2C**), no definitive recurrence or metastasis was identified. However, at 9 months post-surgery (June 2026), he presented earlier than scheduled because he noticed a progressively enlarging mass in the left neck. Contrast-enhanced CT and ultrasonography of the neck revealed a large, irregular, ill-defined solid mass (approximately 60 mm × 39 mm) in the left 101L region, encasing the left common carotid artery, with evidence of tumor thrombus in the left internal jugular vein (**Figure 2D**). No other significant lymphadenopathy was observed in the mediastinum or abdomen. Ultrasound-guided core needle biopsy of the left cervical mass confirmed metastatic squamous cell carcinoma, histologically consistent with the primary esophageal lesion. Based on imaging and pathological findings, a definitive diagnosis of left cervical (101L group) recurrence with venous tumor thrombus was established.

### 2.4 Management of recurrence and follow-up

After the diagnosis of left cervical (101L group) recurrence with internal jugular vein tumor thrombus was confirmed, the patient received concurrent chemoradiotherapy. Radiotherapy was delivered to the supraclavicular lymphatic drainage area as the planning target volume (PTV) at a dose of 50 Gy in 25 fractions (2 Gy per fraction), and to the metastatic 101L lymph node as the planning gross tumor volume for the node (PGTVnd) at a dose of 60 Gy in 30 fractions (2 Gy per fraction). Concurrent chemotherapy consisted of nab-paclitaxel plus carboplatin. After radiotherapy and 4 cycles of concurrent chemotherapy, the metastatic lymph node showed partial response (PR). The patient is currently planned to receive the remaining cycles of chemotherapy and to start sequential immunotherapy with tislelizumab; these treatments have not yet been initiated. At the latest follow-up, the patient was alive with controlled disease and no evidence of new distant metastases.

## 3 Discussion

This case highlights a potential limitation of the current risk-stratification approach for superficial esophageal squamous cell carcinoma. The Japanese Esophageal Society (JES) classification defines SM2 as invasion into the middle third of the submucosa, and current JES/JSGE guidelines recommend esophagectomy for tumors invading beyond 200 μm into the submucosa (4). Despite a tumor infiltration depth of 370 μm-clearly falling within the pT1b (SM2) category and exceeding the absolute indication for endoscopic resection—and the absence of lymphovascular invasion, the patient developed rapid cervical lymph node recurrence within months of undergoing curative-intent surgery. This unfavorable clinical trajectory draws attention to two frequently underestimated determinants of outcome: the tumor infiltration (INF) pattern and the distinct lymphatic anatomy of the middle thoracic esophagus. In selected clinical scenarios, these factors may warrant consideration alongside invasion depth and LVI status in individualized risk assessment. The clinical course also suggests that the small 101L node detected preoperatively may already have represented an occult micrometastasis. Tumor dormancy provides a plausible explanation for the latent interval: disseminated cancer cells can remain quiescent for extended periods before emerging as overt disease, and esophageal dormant cancer stem cells may evade immune elimination through PD-L1 upregulation and CD8+ T cell exclusion (5).

The clinical significance of the INF pattern warrants particular scrutiny. Although depth of invasion and LVI status remain the cornerstones of clinical decision-making, a growing body of evidence has established INFc (diffuse infiltrative type) as an independent risk factor for lymph node metastasis in superficial ESCC (1, 2). The literature on INFb (intermediate type) is less extensive; nevertheless, some studies have suggested a potential association with elevated metastatic risk (3). A recent study of 368 superficial esophageal squamous cell carcinoma (SESCC) patients who underwent ESD demonstrated that INFc was significantly associated with deeper infiltration, lymphovascular invasion, and positive resection margins, and that tumor size, IPCL type B2, and depressed gross morphology were more indicative of INFc-type SESCC (6). In our patient, the tumor exhibited an INFb pattern, characterized by scattered, invasive cell clusters at the tumor front. These cells appear to possess the capacity to infiltrate the lymphatic network through interstitial spaces without forming overt intravascular tumor emboli—a biological feature that offers a possible explanation for why nodal metastasis occurred despite negative LVI on immunohistochemical staining. On the basis of these observations, we advocate that INF classification be routinely reported in post-ESD pathology reports, rather than an optional item. For INFc, it should be regarded as a high-risk signal that warrants aggressive management. For INFb, although the evidence supporting its independent prognostic value is not as robust as that for INFc, its biological behavior suggests that it should not be dismissed—particularly when other risk factors such as mid-thoracic location or suspicious preoperative imaging findings are concurrently present.

In parallel, the anatomical predisposition of the middle thoracic esophagus to cervical metastasis must be fully appreciated. The esophageal submucosal and intermuscular lymphatic networks contain longitudinal communicating branches that run along the length of the esophagus. Notably, the middle thoracic esophagus gives rise to lymphatic channels that ascend along the longitudinal axis, draining directly into the paratracheal and cervical paraesophageal (station 101) lymph nodes (7, 8). Recent evidence from a systematic review and a study of lymph node metastatic patterns in thoracic superficial ESCC indicates that cervical nodes lie within the esophageal longitudinal lymphatic drainage pathways and are primary drainage targets for mid-thoracic tumors, supporting their inclusion within the regional lymphatic territory (9, 10). In this case, the primary tumor was situated 25–26 cm from the incisors, placing it well within the drainage territory of these ascending pathways and providing an anatomical foundation for tumor cell colonization of the left 101L region. This observation suggests that, when planning lymphadenectomy strategies, tumors of the middle thoracic esophagus warrant greater attention to the cervical lymphatic drainage territory compared with tumors located in the lower thoracic segment, where the predominant drainage is toward the abdominal and mediastinal nodes.

The present case also prompts reconsideration of the inherent limitations of two-field lymphadenectomy and the appropriate threshold for selecting three-field dissection. Standard two-field dissection is confined to levels below the thoracic inlet and cannot clear cervical nodal stations such as 101L (11). The optimal extent of lymphadenectomy for thoracic ESCC remains controversial. A recent systematic review and meta-analysis found that three-field lymphadenectomy did not improve overall or disease-free survival compared with two-field lymphadenectomy in unselected patients (12). Similarly, a randomized clinical trial of middle and lower thoracic ESCC showed no long-term survival benefit of three-field over two-field dissection (13). These findings underscore that any potential benefit of three-field dissection is likely confined to selected subgroups, rather than being a universal recommendation. The JES practice guidelines acknowledge that the optimal extent of lymphadenectomy for thoracic esophageal cancer remains a subject of debate, and that cervical nodal stations may be considered part of the regional drainage territory depending on tumor location and stage (14). Importantly, preoperative CT in this patient had already visualized a suspicious lymph node in the 101L region. At that time, however, preoperative tissue diagnosis of this node was not considered, because the tumor was judged to be superficial pT1b (SM2) with negative LVI, and the likelihood of cervical metastasis was therefore considered low. Functional imaging with ¹^8^F-FDG PET-CT was also not performed. It should be emphasized that ¹^8^F-FDG PET-CT is not a novel proposal arising from this single case, but an established component of current staging guidelines. Both the ESMO Clinical Practice Guidelines and the NCCN Guidelines recommend ¹^8^F-FDG PET-CT for patients with esophageal cancer who are candidates for esophagectomy, and explicitly endorse its use in this setting (15, 16). In this patient, ¹^8^F-FDG PET-CT was not performed because the tumor was considered superficial and the risk of metastasis was judged to be low. Retrospectively, this case illustrates that in superficial ESCC—even pT1b (SM2) with negative LVI—a suspicious cervical node on CT should prompt guideline-concordant ¹^8^F-FDG PET-CT before deciding on the extent of lymphadenectomy. The value of ¹^8^F-FDG PET-CT in this specific setting is not merely to detect distant metastases, but to provide functional evidence regarding the malignant potential of an equivocal cervical node, thereby informing whether a three-field dissection should be considered.

We therefore propose that, in patients with superficial ESCC and a cervical node of uncertain significance on conventional imaging, ¹^8^F-FDG PET-CT should be performed in accordance with existing staging guidelines before proceeding to surgery. When ¹^8^F-FDG PET-CT is unavailable or clinical suspicion remains high despite negative functional imaging, individualized three-field dissection—limited to patients with imaging evidence of cervical nodal involvement—may be reasonable in selected cases, although this approach requires validation in prospective studies. Moreover, INFb has not been validated as a criterion for determining the extent of lymphadenectomy. It should not alone justify three-field dissection but should be considered alongside other high-risk features, such as preoperative imaging findings and tumor location.

It is important to distinguish our proposal from prophylactic, unselected three-field lymphadenectomy. Ariyoshi et al. reported that prophylactic supraclavicular lymphadenectomy did not improve prognosis in patients with upper and middle thoracic ESCC (7). However, that study evaluated a prophylactic and unselected approach—routine dissection in all patients regardless of preoperative imaging findings. In contrast, our recommendation is individualized and imaging-triggered: three-field dissection should be considered specifically when preoperative imaging identifies a suspicious cervical node in a patient with high-risk tumor biology, such as pT1b (SM2) with an INFb pattern. These two strategies differ fundamentally in patient selection and clinical intent. The negative results of prophylactic dissection in unselected patients do not preclude a potential benefit of targeted dissection in a carefully selected subgroup with imaging evidence of cervical involvement.

Compared with previously reported recurrence cases following ESD or surgery (17–19), the distinctive value of this case lies in its integrated depiction of the complete trajectory of failure driven by three converging factors: anatomical susceptibility of the middle esophagus, a preoperatively detectable suspicious lymph node that was clinically underappreciated, and a high-risk infiltrative tumor front represented by the INFb pattern. On this basis, we propose that for middle thoracic ESCC with a cervical node detected on preoperative imaging, the patient should be classified as high-risk for recurrence when the pathology shows INFc, or INFb combined with other high-risk features (e.g., mid-thoracic location, suspicious imaging findings). In this context, even in the absence of LVI, individualized consideration of three-field lymphadenectomy and intensified postoperative imaging surveillance are recommended to facilitate early detection and management of occult metastasis. Ultimately, risk assessment for superficial esophageal squamous cell carcinoma must move beyond single-variable determinism toward a multidimensional framework that integrates depth of invasion, LVI status, INF pattern, and tumor location in a holistic manner.

Several limitations of this study should be acknowledged. First, as a single case report, the generalizability of our findings is limited and will require validation in larger cohort studies. Second, the absence of preoperative ¹^8^F-FDG PET-CT precluded functional characterization of the suspicious 101L node, representing a missed opportunity to establish its malignant nature at a functional imaging level prior to surgery. Third, we did not perform high-throughput next-generation sequencing on the recurrent specimen, and thus cannot explore the clonal evolutionary relationship between the primary and recurrent lesions at the molecular level.

## 4 Conclusions

For pT1b (SM2) squamous cell carcinoma of the middle thoracic esophagus with an INFb pattern, the risk of cervical lymph node metastasis remains considerable, even in the absence of LVI. When preoperative imaging identifies cervical lymph nodes of uncertain significance, ¹^8^F-FDG PET-CT should be performed in accordance with current staging guidelines before deciding on the extent of lymphadenectomy, and individualized selection of three-field lymphadenectomy should be considered to minimize the risk of cervical regional recurrence. Rigorous postoperative surveillance using combined cervical and thoracic CT is essential, with particular attention directed to the cervical lymphatic drainage territories, to enable early detection and timely management of occult recurrent metastases.

## Data Availability

The original contributions presented in the study are included in the article/supplementary material. Further inquiries can be directed to the corresponding author.
